# The Difference

**Published:** 1897-09

**Authors:** 


The Difference.—When men argue from opposite premises they can never reach the same conclusion. Our luke warm contemporary of the *Southwestern Medical Record* takes us to task for saying that the failure of the State Medical Association to get their homœopathic-compromise bill through the legislature would prove to be a blessing in disguise. Dr. Red, of the *Southwestern Medical Record* is one of the *homologators*, and of course, does not see the matter as I do. He makes a plea for the "other schools of medicine;" I deny that there are any "schools of mddicine." The *Record* accepts the designation given us by Hahnemann,—confesses that its editor belongs to the "old school" of "allopaths." I repudiate it. There is but one school of medicine; all other so called "schools of medicine" are mere "pathies." There are no "homœopathic physicians;" the term is contradictory; it is an absurdity. Homœopaths are, in no sense, *physicians*, So long as they practice what they pretend to practice,—homœopathy, they are outside the pale of recognition; they are not physicians and have no claim to be considered in medical legislation. But they do not confine themselves to homœopathy; they essay to practice on principles in which they are not versed, and hence they are pretenders and frauds. When they attempt to practice medicine they should be measured by the same medical standard, and made to conform to the same requirements as other medical men are. That is the position of Father Davis, and the position that is exemplified by our code of ethics. We follow Davis; our colleague of the *Southwestern Medical Record* may follow Hahnemann if the company suits him; none of it in ours, if you please.

---

In announcing in our last issue the resignations of Drs. West and Clopton from the Texas Medical College, we neglected to state that Dr. Cerna had been "let out," also, by the regents. Dr. Cerna was not a professor, but an adjunct and lecturer. He is a native Mexican, a very talented men, and a fluent speaker in several languages. Dr. Cerna is the representative of his country, the Republic of Mexico, as consul at Galveston.

